# Protective effect of *Ruellia tuberosa* L. extracts against abnormal expression of hepatic detoxification enzymes in diabetic rats

**DOI:** 10.1039/c8ra03321h

**Published:** 2018-06-13

**Authors:** Wen-Chang Chang, Da-Wei Huang, Jou-An Chen, Yu-Fang Chang, James Swi-Bea Wu, Szu-Chuan Shen

**Affiliations:** Graduate Institute of Food Science and Technology, National Taiwan University P.O. Box 23-14 Taipei 10672 Taiwan; Department of Food and Beverage Management, China University of Science and Technology No.245, Sec. 3, Academia Rd. Taipei 11581 Taiwan; Department of Human Development and Family Studies, National Taiwan Normal University No. 162, Sec. 1, Heping East Rd. Taipei 10610 Taiwan scs@ntnu.edu.tw +886-2-23639635 +886-2-77341437

## Abstract

*Ruellia tuberosa* L. (RTL) has been used as a folk medicine for curing diabetes mellitus in East Asia decades. This study investigated the effect of RTL on hepatic detoxification enzyme expression in diabetic rats. Male Wistar rats were fed a high fat diet (HFD) and intraperitoneally injected with streptozotocin (STZ) to induce diabetes. Subsequently, rats received oral administrations of 100 or 400 mg kg^−1^ body weight RTL extract, in either water (RTLW) or ethanol (RTLE), once a day for 4 weeks. The real-time PCR analyses showed that abnormality of hepatic phase I and II detoxification enzyme expression was observed in diabetic rats. However, both RTLW and RTLE significantly normalized the expression of hepatic phase I detoxification enzymes such as CYP 2E1, and expression of phase II detoxification enzymes such as UGT 1A7 and GST M1 in diabetic rats. Furthermore, we found that fasting serum glucose, hemoglobin A1C (HbA1C) and the area under the curve of oral glucose tolerance test (AUC_OGTT_) levels were significantly reduced in both RTLW and RTLE treated diabetic rats. Moreover, both RTLW and RTLE significantly increased the activity of hepatic anti-oxidative enzymes such as superoxide dismutase (SOD) in diabetic rats. The present study suggests that RTL may ameliorate abnormal hepatic detoxification function *via* alleviating hyperglycemia and enhancing hepatic antioxidant capacity in HFD/STZ-induced diabetic rats.

## Abstract 2

*Ruellia tuberosa* L. (RTL) has been used as a folk medicine for curing diabetes mellitus in East Asia decades.

## Graphical abstract

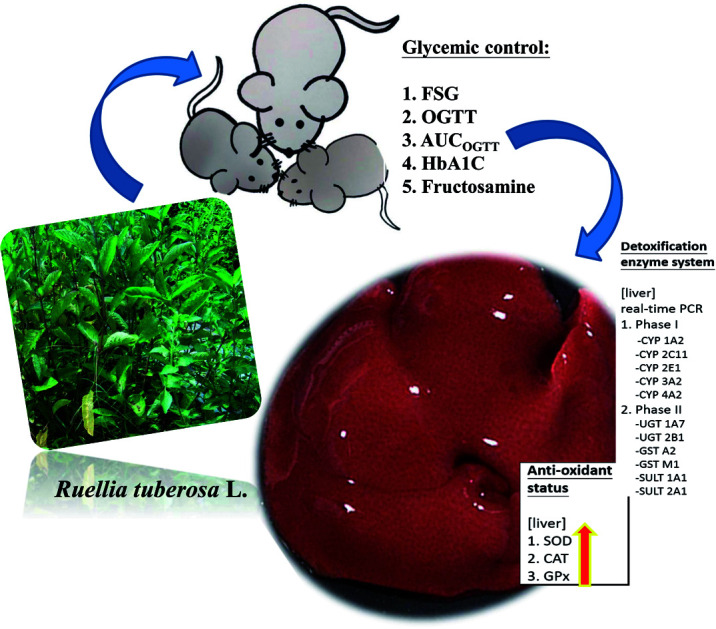

## 1. Introduction

Diabetes mellitus (DM) is a metabolic disease caused by an insulin secretion deficiency or insulin dysfunction, which leads to hyperglycemia. DM is an important chronic disease and it is the 5^th^ leading cause of death in the world currently. Type 1 DM (T1DM) and type 2 DM (T2DM) are the two major types, and 95% of patients with DM have T2DM.^1^ T2DM is characterized by insulin resistance, *i.e.*, reduced insulin sensitivity of cells and the inability of cells to facilitate glucose uptake, which results in abnormally high blood glucose levels.^2^

The liver contains many detoxification enzymes. It is the most important detoxification organ in the body. The hepatic drug metabolism system is divided into two phases, *i.e.*, phase I enzymes and phase II enzymes systems. Phase I enzymes are responsible for functionalization. These enzymes alter functional groups on xenobiotics to improve their hydrophilicity, which either facilitates their excretion or elevates their polarity for proceeding to phase II.^3–5^ Phase II enzymes are responsible for conjugation. These enzymes conjugate phase I-modified xenobiotics with highly polar molecules, which converts them into hydrophilic, inactive compounds that can be excreted into the bile, feces, or urine. High levels of blood sugar may increase oxidative stress in human body and result in the abnormality of detoxification function in the liver of DM patients.^6^ Impairments in detoxification cause accumulations of exogenous or endogenous xenobiotics, which then become toxic in the body.^7–9^ In addition, reactive oxygen species (ROS) are produced during phase I, which can increase the risk of liver injury and reduce the detoxification capacity of the liver.^10^

*Ruellia tuberosa* Linn. (RTL), native to tropical America, is a species of *Ruellia* in the Acanthaceae family. This plant has been used for decades as folk medicine for treating diabetes in East Asia. RTL was reported to possess anti-diabetic, anti-oxidant, and anti-inflammatory activities.^11–13^ The hydro-ethanolic leaf extract of *Ruellia tuberosa* L. possesses abilities to reduce serum phospholipids, triglycerides, total cholesterol, LDL-c and VLDL-c levels, and increase HDL-c level in diabetic rats.^14^ Various polyphenols and flavonoids, such as apigenin, luteolin, 3,5-diglucoside, apigenin-7-*O*-glucuronide, apigenin glucoside, apigenin rutinoside, luteolin glucoside, flavone glycoside and cirsimaritin, cirsimarin, cirsiliol 4-glucoside, sorbifolin, and pedalitin along with betulin, vanillic acid, and indole-3-carboxaldehyde were were reported to be isolated from *Ruellia tuberosa* L.^15^ However, literature is limited regarding the effect of RTL on T2DM. The aim of this study is to investigate the ameliorative effect of RTL on hepatic detoxification enzyme system in a T2DM rat model.

## 2. Materials and methods

### 2.1 Plant materials

The stems and leaves of *Ruellia tuberosa* Linn. (RTL) were collected from the Herb Light farm, Yi-Lan County, Taiwan, in October of 2014.

### 2.2 Preparation of RTL extracts

The plant materials were washed, dried, weighed, sliced, and freeze dried. Each 1 g of dried stem and leaf were extracted with 6 mL of distilled water (RTLW) or 95% ethanol (RTLE) (1 : 6, w/v) individually at 4 °C for 72 h, and then filtered through cheese cloth. The filtrate is filtered twice through Whatman No. 1 filter paper, and then, centrifuged at 4700 × *g* for 20 min. The supernatant is vacuum concentrated using a rotary evaporator below 40 °C. The concentrate was freeze dried into a powder and stored at 80 °C until used. The extraction rate of the RTLW and RTLE were 11.4% and 3.1%, respectively. The appearance of both crude extract powders was brownish green after being freeze dried.

### 2.3 Chemicals and reagents

Bovine serum albumin (BSA), d-(+)-glucose, disodium hydrogen phosphate (Na_2_HPO_4_), ethanol, 4-(2-hydroxyethyl)-1-piperazineethanesulfonic acid (HEPES), mannitol, methanol, pioglitazone hydrochloride (Pio), potassium chloride (KCl), potassium dihydrogen phosphate (KH_2_PO_4_), streptozotocin (STZ), sodium chloride (NaCl), sodium citrate dehydrate (C_6_H_5_O_7_Na_3_·2H_2_O), and sucrose, were purchased from Sigma (St Louis, MO, USA). 1,4-Dithiothreitol (DTT) was obtained from Merk KGaA (Darmstadt, Germany). The protease inhibitor cocktail tablet was purchased from Roche (Mannheim, Germany). The Bio-Rad protein assay dye reagent was obtained from Bio-Rad Laboratories (Richmond, VA, USA). All of the chemicals used in this study are of analytical grade.

### 2.4 Animal experimental procedure

Male Wistar rats (4 weeks-old) were obtained from the National Laboratory Animal Center, Taipei, Taiwan. The room conditions and treatment procedures were in accordance with the National Institutes of Health Guide for the Care and Use of Laboratory Animals, and all of the protocols were approved by the Institutional Animal Care and Use Committee of National Taiwan Normal University, Taipei, Taiwan (approval no. 103042, 23 December 2014). The rats were maintained in standard laboratory conditions (22 ± 1 °C and a 12 h light/12 h dark cycle) with free access to food and water. After one week adaptation, the rats were fed with high fat (unsalted butter) diet (HFD, 60% calories from fat) for 4 weeks. In the 5^th^ and 6^th^ weeks, the STZ (28 and 15 mg kg^−1^ body weight, respectively, is dissolved in 0.1 M sodium citrate buffer at pH 4.5) is intraperitoneally injected into each HFD rat to induce diabetes. After the STZ injection, rats were supplied with drinking water containing 5% sucrose for 48 hours, in order to reduce early death due to insulin discharge from partially injured pancreatic islets. Seventy-two hours later, rats were checked for hyperglycemia. For the animal experimental design, the rats were divided into 7 groups (each contains 6 rats): Group 1 consists of rats fed a normal diet for 10 weeks; Group 2 consists of diabetic rats fed an HFD (60% calories from fat) for 10 weeks as the negative control; Group 3 consists of diabetic rats fed an HFD for 10 weeks and gavaged with Pio (30 mg kg^−1^ body weight) daily during the last 4 weeks as the positive control; Groups 4 and 5 consisted of diabetic rats fed an HFD for 10 weeks and gavaged with RTLW (100 or 400 mg kg^−1^ body weight, respectively) daily during the last 4 weeks; Groups 6 and 7 consisted of diabetic rats fed an HFD for 10 weeks and gavaged with RTLE (100 or 400 mg kg^−1^ body weight, respectively) daily during the last 4 weeks of this 10 week period. The rats were scarified at the end of the experiment, blood samples were collected, and the biochemical analysis was conducted. The livers were stored at −80 °C for further analysis.

### 2.5 Blood sample preparation

Blood samples were collected and allowed to clot for 30 min at room temperature, and then, centrifuged at 3000 × *g* for 10 min twice to obtain the serum, which is stored at −80 °C till they was used.

### 2.6 Oral glucose tolerance test

Oral glucose tolerance testing (OGTT) was performed on rats in all groups after an overnight fasting. All animals were orally administered 1.5 g of glucose per kg body weight. Blood was sampled from the tail vessels of conscious animals before (*t* = 0) and 30, 60, 90, and 120 min after glucose administration. The samples were allowed to clot for 30 min, and then, centrifuged (4 °C, 3000 × *g*, 20 min) to obtain the serum. Glucose concentration was determined using a glucose enzymatic kit (Crumlin Co., Antrim, UK). The obtained glucose concentration values were plotted against time to provide a curve showing the changes in glucose levels over time and expressed as an integrated area under the curve for glucose (AUC_OGTT_).

### 2.7 Homogeneous solution from rat liver tissue

Liver tissue (0.05 g) was homogenized with 0.25 mL of 50 mM sodium phosphate buffer solution (pH 7.4) in an ice water bath using a homogenizer (1000 rpm, 15 s), and then, centrifuged (10 000 × *g*, 10 min, 4 °C) to acquire the supernatant for further experiment.

### 2.8 mRNA extraction and quantitative real-time PCR assay

The total RNAs were isolated from the liver tissues using the Direct-zol™ RNA MiniPrep Kit (R2053) (Zymo Research Corporation, Irvine, CA, USA) according to the manufacturer's protocol. The concentration and purity of the extracted RNA were checked spectrophotometrically by measuring the 260/280 absorption ratios. The primers were designed online by the Primer-Blast of National Center for Biotechnology Information (NCBI) and synthesized by Genomics (New Taipei City, Taiwan), as shown in Table 1. The expression levels of various detoxification phase I (CYP1A2, CYP2C11, CYP2E1, CYP3A2 and CYP4A2) and phase II (UGT1A7, UGT2B1, GSTA2, GSTM1, SULT1A1 and SULT2A1) genes were also determined. Then, 1 μg of total RNA were reverse transcribed into cDNA using a high capacity cDNA reverse transcription kit (Applied Biosystems, Foster City, CA, USA), and gene expressions were determined by mixing 0.5 μL cDNA, 10 μL SYBR® Green PCR master mix (Applied Biosystems, Foster City, CA, USA), and appropriate primer pairs in a final volume of 10 μL. PCR was carried out with an initial cycle of 95 °C for 10 min, followed by 40 cycles, each consisting of 95 °C, 15 s and 60 °C, 1 min. The β-actin gene was amplified as the internal control. Each sample was amplified in triplicate, and the differences in the mRNA expression were calculated using the ΔΔC_T_ method.

**Table 1:** Primer sequences for RT-PCR

CYP 1A2	(Forward)	5′-CATCCCCCACAGCACAACGA-3′
	(Reverse)	5′-GGTAAGAAACCGCTCTGGGC-3′
CYP 2C11	(Forward)	5′-GGGCCTTGGAGTCATTTTTAGCA-3′
	(Reverse)	5′-AGCCCAGGATAAAGGTGGGA-3′
CYP 2E1	(Forward)	5′-TGGAGAAGGAAAAACACAGCCAAGA-3′
	(Reverse)	5′-CTTGGCCCAATAACCCTGTCA-3′
CYP 3A2	(Forward)	5′-GGATCTTCACAAAGGCAGTGTC-3′
	(Reverse)	5′-CCATCACAGACCTTGCCAACT-3′
CYP 4A2	(Forward)	5′-AGATCAGATCCAAAGCCTTATCA-3′
	(Reverse)	5′-CTGGTCATCAAGCTTCTCCCA-3′
UGT 1A7	(Forward)	5′-CCGATGATGAGCCTCTGGAC-3′
	(Reverse)	5′-CACGTCCAAGGAGTGGTACT-3′
UGT 2B1	(Forward)	5′-AGAAGTCCTGGAGTCAGTTTTACA-3′
	(Reverse)	5′-AAATTCTTCCATTTCCCTAGGCAGT-3′
GST A2	(Forward)	5′-GGAGAGAGCCCTGATTGACA-3′
	(Reverse)	5′-TTGGCCATGGCTCTTCAACA-3′
GST M1	(Forward)	5′-GTTTGCAGGGGACAAGGTCAC-3′
	(Reverse)	5′-GGCCAACTTCGAAAATATAGGTGT-3′
SULT 1A1	(Forward)	5′-GCCCGAAATGCAAAGGATGT-3′
	(Reverse)	5′-ACCACGACCCATAGGACACT-3′
SULT 2A1	(Forward)	5′-TCCAAGGCCAAGGTGATCTAT-3′
	(Reverse)	5′-CACGGATGTGCTCAAACCAT-3′
β-actin	(Forward)	5′-ACAACCTTCTTGCAGCTCCTC-3′
	(Reverse)	5′-CTGACCCATACCCACCATCAC-3′

### 2.9 Biochemical measurements

The blood hemoglobin A1C (HbA1C), serum fructosamine, and hepatic anti-oxidative enzyme includes catalase (CAT), glutathione peroxidase (GPx) and superoxide dismutase (SOD) activity are determined using enzyme-linked immunosorbent assay (ELISA) kits which are purchased from the Cayman Chemical Company (Ann Arbor, Michigan, USA). Biochemical analyses were performed according to the manufacturer's protocols.

### 2.10 Statistical analysis

Results are presented as the mean ± standard deviation (SD), which is analyzed using one-way ANOVA and Duncan's new multiple range tests. All comparisons are made relative to the normal group, where *p* < 0.05 is considered to be significant.

## 3. Results and discussion

### 3.1 Effect of RTLW and RTLE on phase I enzymes in liver of HFD/STZ rats

**Fig. 1:**
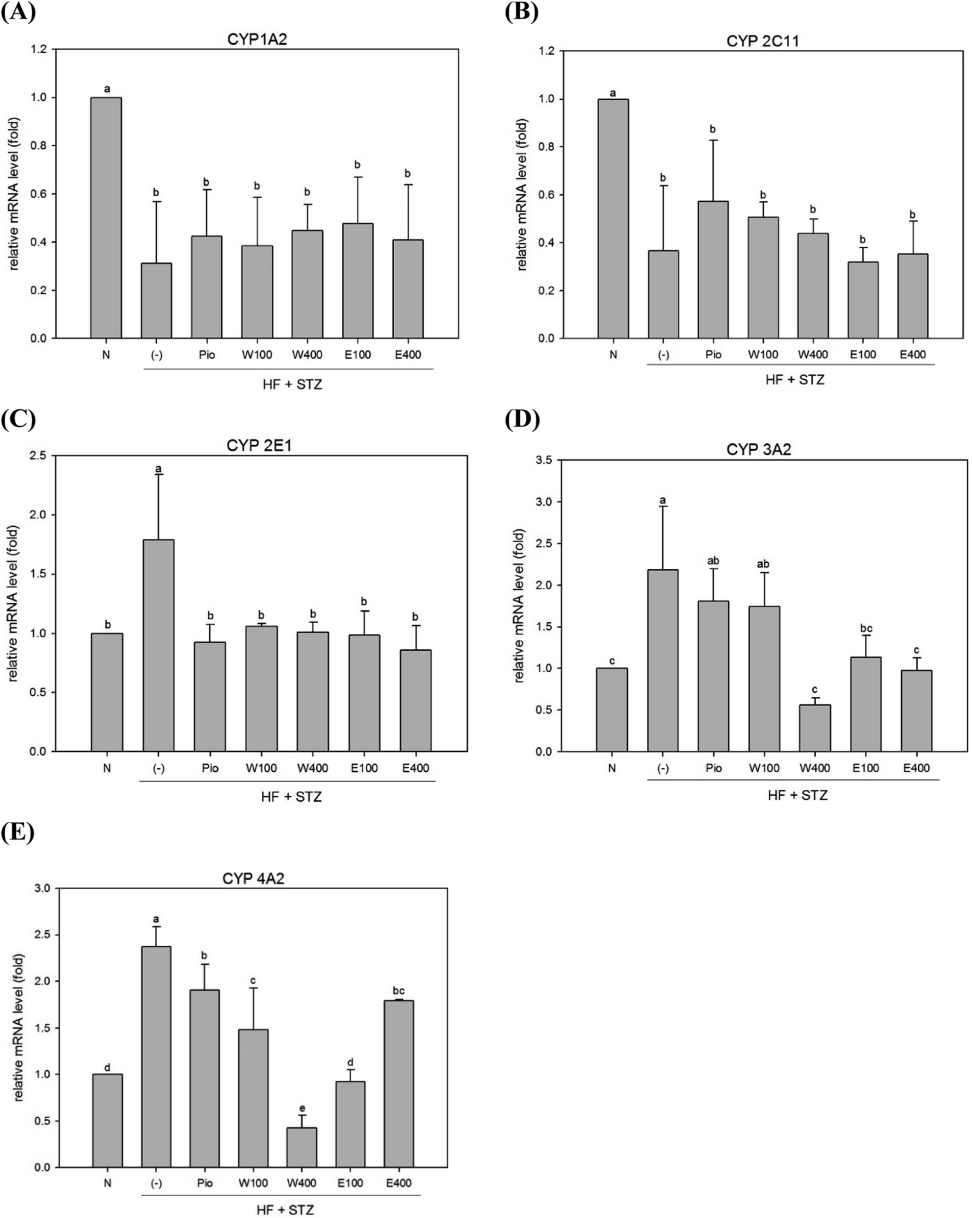
The effect of RTLW, RTLE and pioglitazone on hepatic phase I detoxification relative mRNA level in HFD/STZ-induced diabetic rats. RTL = *Ruellia tuberosa* L.; RTLW = *Ruellia tuberosa* L. water extract; RTLE = *Ruellia tuberosa* L. ethanol extract; HFD = high fat diet; STZ = streptozotocin. N = Normal diet; DM = high fat diet (HFD) (60% fat) + STZ intraperitoneal injection; DM + Pio = HFD (60% fat) + STZ intraperitoneal injection + pioglitazone (30 mg kg^−1^ B.W.); DM + W100 = HFD (60% fat) + STZ intraperitoneal injection + RTLW (100 mg kg^−1^ B.W.); DM + W400 = HFD (60% fat) + STZ intraperitoneal injection + RTLW (400 mg kg^−1^ B.W.); DM + E100 = HFD (60% fat) + STZ intraperitoneal injection + RTLE (100 mg kg^−1^ B.W.); DM + E400 = HFD (60% fat) + STZ intraperitoneal injection + RTLE (400 mg kg^−1^ B.W.). a–e letters are significantly different from all samples tested (*p* < 0.05). Each value is means ± SD, *n* = 6 per group.

The high oxidative stress caused by hyperglycemia was considered to be associated with abnormal detoxification function in liver of diabetes.^6^ Dysfunction of the hepatic detoxification enzyme system can result in either incomplete or excessive removal of xenobiotics from the body.^16^ Hepatic detoxification is assured when the expression levels of detoxification enzymes are restored to normal levels in liver.^17^ The majority of evidence has indicated that the CYP system appears to be the first step in hepatocytes detoxification. The CYP 1A2 enzyme, which accounts for 13% of the total amount of hepatic CYP enzymes, can metabolize and discharge 15% of the drugs present in the human body.^18^ As shown in Fig. 1A, the expression of hepatic CYP 1A2 enzyme was significantly reduced in HFD/STZ-induced diabetic rats (0.31-fold of the level in normal rats; *p* < 0.05). After administering RTLW and RTLE for 4 weeks, no significant changes were found in hepatic CYP 1A2 expression in diabetic rats (Fig. 1A).

The CYP 2C family can metabolize over half of the most common drugs.^19^ In this study, hepatic CYP 2C11 mRNA expression was significantly reduced in diabetic rats (Fig. 1B; *p* < 0.05). The level of CYP 2C11 did not significantly change after RTLW and RTLE treatment in diabetic rats (Fig. 1B). CYP 2E1 plays an important role in the metabolism of chemicals and carcinogens.^20^ Previous studies reported that CYP 2E1 expression was significantly elevated in DM patients^21^ and in STZ induced diabetic rats.^22^ The results from the present study indicated that expression of hepatic CYP 2E1 mRNA was significantly elevated in diabetic rats (Fig. 1C; *p* < 0.05), whereas RTLW, RTLE and Pio-treatments reduced hepatic CYP 2E1 expression in diabetic rats and normalized the levels to that of normal rats (Fig. 1C; *p* < 0.05).

Malekinejad *et al.* reported that the expression of CYP 3A2 mRNA was markedly elevated in the liver of STZ-induced diabetic rats.^17^ In contrast, Vornoli *et al.* found no significant change on protein levels of hepatic CYP 3A2 in HFD/STZ treated Wistar rats.^16^Fig. 1D shows that expression of hepatic CYP 3A2 mRNA was significantly elevated in HFD/STZ-induced diabetic rats. Furthermore, the administration of RTLW (W400) or RTLE (E100, E400) were found to suppress hepatic CYP 3A2 overexpression in diabetic rats and restored it approximates to that of normal rats (Fig. 1D; *p* < 0.05).

Vornoli *et al.* revealed that the expression levels of hepatic CYP 4A1 and CYP 4A2 mRNA were significantly elevated in HFD/STZ-induced diabetic rats.^16^ Our results also showed that expression of hepatic CYP 4A2 mRNA was enhanced in HFD/STZ-induced diabetic rats (Fig. 1E; *p* < 0.05), which is consistent with previous study.^16^ In addition, the administration of RTLW and RTLE significantly decreased hepatic CYP 4A2 mRNA expression in diabetic rats and normalized them to that of normal rats (Fig. 1E; *p* < 0.05).

### 3.2 Effect of RTLW and RTLE on phase II enzymes in the livers of HFD/STZ rats

**Fig. 2:**
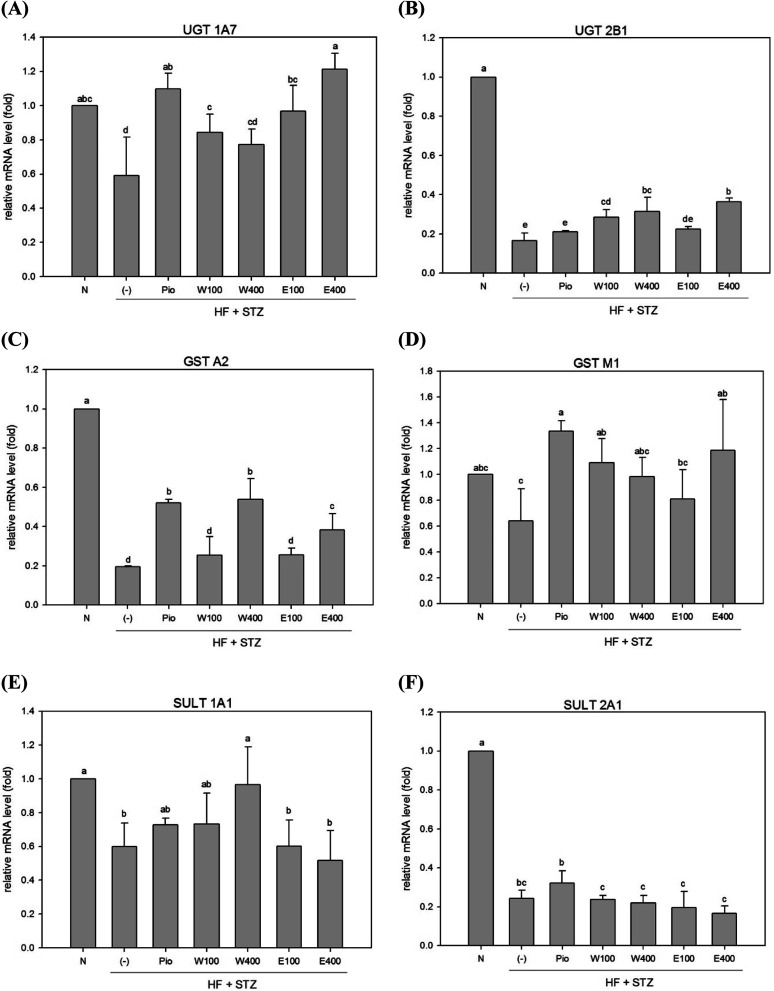
The effect of RTLW, RTLE and pioglitazone on hepatic phase II detoxification relative mRNA level in HFD/STZ-induced diabetic rats. RTL = *Ruellia tuberosa* L.; RTLW = *Ruellia tuberosa* L. water extract; RTLE = *Ruellia tuberosa* L. ethanol extract; HFD = high fat diet; STZ = streptozotocin. N = Normal diet; DM = high fat diet (HFD) (60% fat) + STZ intraperitoneal injection; DM + Pio = HFD (60% fat) + STZ intraperitoneal injection + pioglitazone (30 mg kg^−1^ B.W.); DM + W100 = HFD (60% fat) + STZ intraperitoneal injection + RTLW (100 mg kg^−1^ B.W.); DM + W400 = HFD (60% fat) + STZ intraperitoneal injection + RTLW (400 mg kg^−1^ B.W.); DM + E100 = HFD (60% fat) + STZ intraperitoneal injection + RTLE (100 mg kg^−1^ B.W.); DM + E400 = HFD (60% fat) + STZ intraperitoneal injection + RTLE (400 mg kg^−1^ B.W.). a–e letters are significantly different from all samples tested (*p* < 0.05). Each value is means ± SD, *n* = 6 per group.

**Fig. 3:**
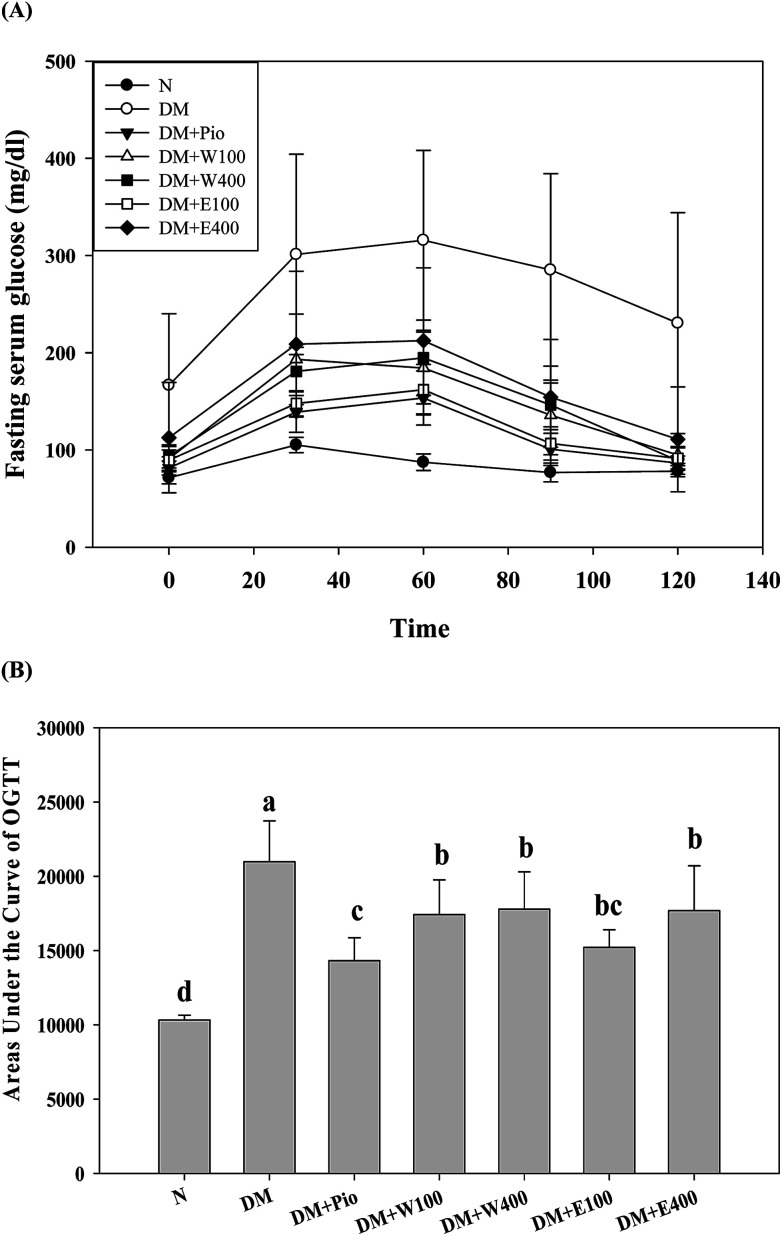
(A) Oral glucose tolerance test (OGTT) and (B) the areas under the curve of OGTT (AUC_OGTT_) of HFD/STZ-induced diabetic rats fed with RTLW, RTLE and pioglitazone for 4 weeks. RTL = *Ruellia tuberosa* L.; RTLW = *Ruellia tuberosa* L. water extract; RTLE = *Ruellia tuberosa* L. ethanol extract; OGTT = oral glucose tolerance test; AUC_OGTT_ = the areas under the curve of OGTT; HFD = high fat diet; STZ = streptozotocin. N = Normal diet; DM = HFD (60% fat) + STZ intraperitoneal injection; DM + Pio = HFD (60% fat) + STZ intraperitoneal injection + pioglitazone (30 mg kg^−1^ B.W.); DM + W100 = HFD (60% fat) + STZ intraperitoneal injection + RTLW (100 mg kg^−1^ B.W.); DM + W400 = HFD (60% fat) + STZ intraperitoneal injection + RTLW (400 mg kg^−1^ B.W.); DM + E100 = HFD (60% fat) + STZ intraperitoneal injection + RTLE (100 mg kg^−1^ B.W.); DM + E400 = HFD (60% fat) + STZ intraperitoneal injection + RTLE (400 mg kg^−1^ B.W.). * indicates a significant difference (*p* < 0.05) compared with the normal group at the same time point. Each value is means ± SD, *n* = 6 per group.

In contrast to phase I enzymes system, far less is known about modulation of phase II enzymes in diabetes. The UGT enzyme family performs glucuronide conjugation, which is the primary function of the phase II detoxification enzyme system. It was previously reported that the mRNA and protein levels of UGTs 1A1, 1A6, 1A7, 2B1, and 1A9 were reduced in livers of obese rats and mice.^23–25^ Our results revealed that the expression levels of hepatic UGT 1A7 and UGT 2B1 mRNAs were significantly decreased in HFD/STZ-induced diabetic rats (Fig. 2A and 3B, *p* < 0.05). However, administering with RTLW (W100, W400) or RTLE (E100) increased hepatic UGT 1A7 mRNA expression in diabetic rats and normalized levels to that of normal rats (Fig. 2A, *p* < 0.05).

Another component of hepatic phase II detoxification is the glutathione (GSH)-related antioxidant system. The GST enzyme catalyzes the conjugation of GSH to xenobiotics in the liver. The GSH conjugation reaction in the liver is affected by GST expression, GSH levels, and glutathione synthetase (GSS) activity. Studies of hepatic GST activity during diabetes are inconclusive; with both increased and decreased GST activities being reported in STZ-induced diabetic rats.^26,27^ The present study revealed that expression of hepatic GST A2 and GST M1 mRNAs were significantly reduced in diabetic rats (Fig. 2C and D; *p* < 0.05). However, high-dose RTLW and RTLE extracts (W400, E400) significantly increased expression of hepatic GST A2 mRNA in diabetic rats (Fig. 2C, *p* < 0.05). Moreover, both RTLW and RTLE significantly enhanced expression of hepatic GST M1 in diabetic rats and restored them approximate to that found in normal rats (Fig. 2D).

The SULT enzyme metabolizes endogenous hormones in addition to at least 1/4 of the therapeutic medicines present in the body.^25^ Our results showed that the expression of hepatic SULT 1A1 and SULT 2A1 mRNAs in diabetic rats were dramatically reduced compared with normal rats (Fig. 2E; *p* < 0.05). However, administering with RTLW (W400) elevated the expression of hepatic SULT 1A1 mRNA in diabetic rats and normalized levels to that of normal rats (Fig. 2E; *p* < 0.05).

The above findings demonstrated that RTLW and RTLE may normalize hepatic phase I enzymes and phase II enzymes expressions in HFD/STZ-induced diabetic rats.

### 3.3 Effect of RTLW and RTLE on glucose tolerance in HFD/STZ rats

The long-term excessive intake of a high-calorie diet is considered to be related to many metabolic symptoms and diseases, such as obesity, T2DM, fatty liver, *etc.* Oral glucose tolerance testing (OGTT) is one way to determine a clinical diagnosis of DM. As shown in Fig. 3A, the initial serum glucose levels in the HFD/STZ-induced diabetic rats were significantly higher than those in the RTL-treatments rats (W100, W400, E100, and E400 groups) and the normal rats (*p* < 0.05). At 30, 60, 90, and 120 min after glucose administration, the HFD/STZ-induced diabetic rats showed a much higher increment in serum glucose levels compared with the other groups in the OGTT. Moreover, the diabetic rats that orally received RTLW and RTLE extracts showed significantly lower serum glucose levels (*p* < 0.05) compared with the serum glucose levels in the diabetic rats and restored to levels similar to those of rats fed normal diets at 120 min (Fig. 3A; *p* < 0.05), indicating the ameliorative ability of RTLW and RTLE on maintaining serum glucose homeostasis. Furthermore, we observed that the AUC_OGTT_ in diabetic rats (20 982.0 ± 2742.1) was significantly higher than that of normal rats (10 322.5 ± 325.9; *p* < 0.05; Fig. 3B). The high area under the curve of the OGTT (AUC_OGTT_) indicates poor glucose tolerance in mammalian species. Previous studies have indicated that a HFD leads to insulin resistance, impaired glucose tolerance, and reduced glucose uptake capacity in rats.^28–33^ The results from present study indicated RTLW and RTLE may improve serum glucose homeostasis and glucose tolerance in HFD/STZ-induced diabetic rats.

### 3.4 Effect of RTLW and RTLE on serum glucose index in HFD/STZ rats

In the human body, glucose is the most abundant simple sugar and lysine is the most abundant primary amino acid. Glucose can react with lysine to form glucosylamine (Schiff base) within minutes; this compound can be further converted to fructosamine (a type of ketoamine), which subsequently forms an irreversible advanced glycation end product.^34^ Fructosamine promotes oxidative stress and inflammation *in vivo*.^35^ The level of fructosamine reflects the level of blood glucose in the body over the last 1 to 3 weeks. Hemoglobin is a protein in red blood cells that carries oxygen to tissues and cells. Glucose may react with the N-terminus of the β-chain of hemoglobin to form glycosylated hemoglobin (or called hemoglobin A1C, HbA1C). The HbA1C level reflects the level of hyperglycemia in the body over the last 2 to 3 months. The results of this study revealed that STZ/HFD significantly increased the blood fructosamine and HbA1C contents in rats; however, both RTLW and RTLE significantly reduced these contents in diabetic rats (Table 2; *p* < 0.05). We speculate that RTLW and RTLE could alleviate hyperglycemia and ameliorate blood glucose homeostasis *via* suppressing insulin resistance in STZ/HFD-induced diabetic rats. Methanolic extract of RTL contains flavonoids includes luteolin, 6-hydroxyluteolin-7-alpha-glucoside and its glycosides, which have known antioxidant properties, may be thought to involve in the improvement of insulin resistance in diabetic rats.^36^

**Table 2:** Effect of RTLW, RTLE and pioglitazone on glycemic index in HFD/STZ-induced diabetic rats for 4 weeks^a^

	N	DM	DM + Pio	DM + W100	DM + W400	DM + E100	DM + E400
Fasting serum glucose (mg dL^−1^)	72.67 ± 9.63^b^	152.25 ± 52.27^a^	99.67 ± 10.01^b^	98.80 ± 26.31^b^	90.83 ± 34.66^b^	96.83 ± 28.51^b^	107.00 ± 27.08^b^
HbA1C (%)	4.55 ± 0.26^c^	7.37 ± 0.70^a^	5.20 ± 0.29^bc^	6.10 ± 0.69^b^	5.78 ± 0.19^b^	5.72 ± 0.15^b^	5.95 ± 1.48^b^
Serum fructosamine (μmol L^−1^)	178 ± 13^b^	257 ± 22^a^	193 ± 19^b^	204 ± 8^b^	193 ± 23^b^	201 ± 85^b^	204 ± 18^b^

a RTL = *Ruellia tuberosa* L.; RTLW = *Ruellia tuberosa* L. water extract; RTLE = *Ruellia tuberosa* L. ethanol extract; HFD = high fat diet; STZ = streptozotocin; HbA1C = hemoglobin A1C. N = normal diet; DM = HFD (60% fat) + STZ intraperitoneal injection; DM + Pio = HFD (60% fat) + STZ intraperitoneal injection + pioglitazone (30 mg kg^−1^ B.W.); DM + W100 = HFD (60% fat) + STZ intraperitoneal injection + RTLW (100 mg kg^−1^ B.W.); DM + W400 = HFD (60% fat) + STZ intraperitoneal injection + RTLW (400 mg kg^−1^ B.W.); DM + E100 = HFD (60% fat) + STZ intraperitoneal injection + RTLE (100 mg kg^−1^ B.W.); DM + E400 = HFD (60% fat) + STZ intraperitoneal injection + RTLE (400 mg kg^−1^ B.W.). a–c letters in the same row are significantly different from all samples tested (*p* < 0.05). Each value is means ± SD, *n* = 6.

### 3.5 Effect of RTLW and RTLE on hepatic anti-oxidative enzymes capacity in HFD/STZ rats

Increased oxidative stress is an important risk factor for progression of DM and hepatic detoxification impairment.^37^ Previous studies found that high blood sugar may lead to the generation of excessive free radicals and adversely reduce the activity of antioxidant enzymes.^38–40^ Consequently, patients with DM have reduced antioxidant capacity.^41,42^ High concentrations of free fatty acids may stimulate the production of superoxide anions, hydrogen peroxide, and H_2_O_2_, which results in high oxidative stress levels in the liver.^43,44^ In addition, ROS are released during the phase I metabolic process, which viciously contributes to the development of DM.^10^Table 3 shows the effect of RTLW and RTLE treatments on hepatic antioxidant capacity in HFD/STZ-induced diabetic rats. The capacity of hepatic anti-oxidative enzymes, including SOD, CAT, and GPx, were significantly reduced in diabetic rats compared to normal rats (Table 3, *p* < 0.05). The administration of RTLW or RTLE increased hepatic SOD capacity in diabetic rats as a dose-dependent manner (Table 3, *p* < 0.05). In addition, RTLE (E100, E400) increased hepatic CAT capacity in diabetic rats, and high doses of RTLW (W400) or RTLE (E400) significantly elevated hepatic GPx capacity in diabetic rats (Table 3, *p* < 0.05). It was hypothesized that the enhancing of hepatic antioxidant capacity comprised a key mechanism underlying the protective activity of RTLW and RTLE on impairment of detoxification function in liver of diabetic rats.

**Table 3:** Hepatic anti-oxidative enzymes capacity of HFD/STZ-induced diabetic rats fed with RTLW, RTLE and pioglitazone for 4 weeks^a^

(U mg^−1^ protein)	N	DM	DM + Pio	DM + W100	DM + W400	DM + E100	DM + E400
SOD	58.56 ± 6.25^a^	36.67 ± 13.65^b^	63.93 ± 18.99^a^	59.52 ± 8.12^a^	61.24 ± 5.84^a^	50.97 ± 5.46^a^	55.85 ± 10.16^a^
CAT	4112.28 ± 731.38^ab^	3247.66 ± 171.65^c^	4233.44 ± 744.2^ab^	3280.55 ± 672.4^bc^	3655.25 ± 656.5^abc^	4050.88 ± 285.61^ab^	4520.69 ± 659.39^a^
GPx	735.61 ± 44.55^a^	429.99 ± 86.7^d^	546.33 ± 60.67^bc^	511.99 ± 41.74^bcd^	561.45 ± 45.8^bc^	473.2 ± 63.71^cd^	576.04 ± 74.22^b^

a SOD = superoxide dismutase; CAT = catalase; Px = glutathione peroxidase; RTL = *Ruellia tuberosa* L.; RTLW = *Ruellia tuberosa* L. water extract; RTLE = *Ruellia tuberosa* L. ethanol extract; HFD = high fat diet; STZ = streptozotocin. N = Normal diet; DM = HFD (60% fat) + STZ intraperitoneal injection; DM + Pio = HFD (60% fat) + STZ intraperitoneal injection + pioglitazone (30 mg kg^−1^ B.W.); DM + W100 = HFD (60% fat) + STZ intraperitoneal injection + RTLW (100 mg kg^−1^ B.W.); DM + W400 = HFD (60% fat) + STZ intraperitoneal injection + RTLW (400 mg kg^−1^ B.W.); DM + E100 = HFD (60% fat) + STZ intraperitoneal injection + RTLE (100 mg kg^−1^ B.W.); DM + E400 = HFD (60% fat) + STZ intraperitoneal injection + RTLE (400 mg kg^−1^ B.W.). a–d letters in the same row are significantly different from all samples tested (*p* < 0.05). Each value is means ± SD, *n* = 6.

The administration of RTLW and RTLE down-regulated the hepatic phase I detoxification enzyme related mRNA expression, including the CYP 2E1, CYP 3A2 and CYP 4A2. RTLW and RTLE also up-regulated the hepatic phase II detoxification enzyme related mRNA expression, including UGT 1A7, UGT 2B1, GST A2, GST M1 and SULT 1A1. RTLW and RTLE significantly reduced the values of the area under the curve for glucose in an oral glucose tolerance test and increased the capacity of hepatic antioxidant enzymes in HFD rats. Based on the above-mentioned results, high dose RTLW (W400) exhibited a better ability than others on alleviating abnormal serum glucose level and expression of hepatic detoxification enzymes in diabetic rats.

## 4. Conclusion

**Fig. 4:**
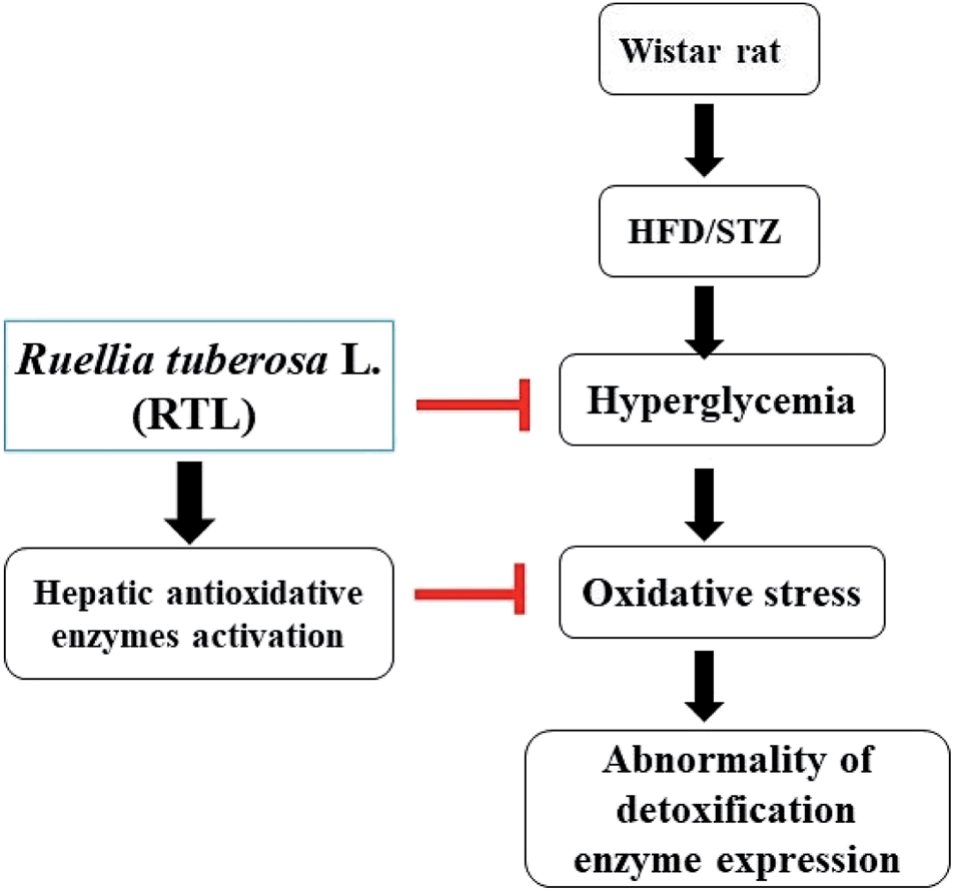
Postulated mechanism for RTL on alleviating hyperglycemia and hepatic detoxification enzyme expression abnormality *via* inhibiting insulin resistance and increasing hepatic anti-oxidative enzyme activities in HFD/STZ-induced diabetic rats.

The present study demonstrated that RTLW and RTLE may alleviate hyperglycemia and enhance hepatic antioxidant capacity, thus suppress the generation of oxidative stress and subsequently normalize hepatic detoxification enzymes expression in HFD/STZ-induced diabetic rats. A postulated mechanism is shown in Fig. 4. Our findings suggested that RTL possesses beneficial potential for becoming a complementary medicine on preventing hyperglycemia and abnormal detoxification function in diabetes mellitus. Further investigation on the purification and identification of active compounds in RTL is currently on the way in our laboratory.

## Conflicts of interest

All the authors declare no conflicts of interest.

## Supplementary Material

## References

[cit1] American Diabetes Association (2015). Standards of medical care in diabetes-2015. Diabetes Care.

[cit2] Jiang Y. D., Chang C. H., Tai T. Y., Chen J. F., Chuang L. M. (2012). Incidence and prevalence rates of diabetes mellitus in Taiwan: analysis of the 2000-2009 Nationwide Health Insurance database. J. Formosan Med. Assoc..

[cit3] Liska D. J. (1998). The detoxification enzyme systems. Altern. Med. Rev..

[cit4] Hines R. N., McCarver D. G. (2002). The ontogeny of human drug-metabolizing enzymes: phase I oxidative enzymes. J. Pharmacol. Exp. Ther..

[cit5] McCarver D. G., Hines R. N. (2002). The ontogeny of human drug-metabolizing enzymes: phase II conjugation enzymes and regulatory mechanisms. J. Pharmacol. Exp. Ther..

[cit6] Rösen P., Nawroth P. P., King G., Möller W., Tritschler H. J., Packer L. (2001). The role of oxidative stress in the onset and progression of diabetes and its complications: a summary of a Congress Series sponsored by UNESCO-MCBN, the American Diabetes Association and the German Diabetes Society. Diabetes/Metab. Res. Rev..

[cit7] Sindhu R. K., Koo J. R., Sindhu K. K., Ehdaie A., Farmand F., Roberts C. K. (2006). Differential regulation of hepatic cytochrome P450 monoxygenases in streptozotocin-induced diabetic rats. Free Radical Res..

[cit8] Merrell M. D., Cherrington N. J. (2011). Drug metabolism alterations in nonalcoholic fatty liver disease. Drug Metab. Rev..

[cit9] Naik A., Belič A., Zanger U. M., Rozman D. (2013). Molecular interactions between NAFLD and xenobiotic metabolism. Front. Genet..

[cit10] Limón-Pacheco J., Gonsebatt M. E. (2009). The role of antioxidants and antioxidant-related enzymes in protective responses to environmentally induced oxidative stress. Mutat. Res..

[cit11] Chen F. A., Wu A. B., Shieh P., Kuo D. H., Hsieh C. Y. (2006). Evaluation of the antioxidant of *Ruellia tuberosa*. Food Chem..

[cit12] Chothani D. L., Patel M. B., Mishra S. H., Vaghasiya H. U. (2010). Review on *Ruellia tuberosa* (Cracker plant). Pharmacogn. J..

[cit13] Rajan M., Kumar V. K., Kumar P. S., Swathi K. R., Haritha S. (2012). Antidiabetic, antihyperlipidaemic and hepatoprotective activity of methanolic extract of *Ruellia tuberosa* Linn. leaves in normal and alloxan induced diabetic rats. J. Chem. Pharm. Res..

[cit14] Ananthakrishnan M., Doss V. A. (2013). Effect of 50% hydro-ethanolic leaf extracts of *Ruellia tuberosa* L. And *dipteracanthus patulus* (jacq.) on lipid profile in alloxan induced diabetic rats. Int. J. Prev. Med..

[cit15] Wulan D. R., Utomo E. P., Mahdi C. (2015). Antidiabetic activity of *Ruellia tuberosa* L., role of α-amylase inhibitor: *in silico*, *in vitro*, and *in vivo* approaches. Biochem. Res. Int..

[cit16] Vornoli A., Pozzo L., Della Croce C. M., Gervasi P. G., Longo V. (2014). Drug metabolism enzymes in a steatotic model of rat treated with a high fat diet and a low dose of streptozotocin. Food Chem. Toxicol..

[cit17] Malekinejad H., Rezabakhsh A., Rahmani F., Hobbenaghi R. (2012). Silymarin regulates the cytochrome P450 3A2 and glutathione peroxides in the liver of streptozotocin-induced diabetic rats. Phytomedicine.

[cit18] Zhou S. F., Chan E., Zhou Z. W., Xue C. C., Lai X., Duan W. (2009). Insights into the structure, function, and regulation of human cytochrome P450 1A2. Curr. Drug Metab..

[cit19] Nebert D. W., Russell D. W. (2002). Clinical importance of the cytochromes P450. Lancet.

[cit20] Lu Y., Cederbaum A. I. (2008). CYP2E1 and oxidative liver injury by alcohol. Free Radical Biol. Med..

[cit21] Gomez-Lechon M. J., Jover R., Donato M. T. (2009). Cytochrome P450 and steatosis. Curr. Drug Metab..

[cit22] Wang T., Shankar K., Ronis M. J., Mehendale H. M. (2000). Potentiation of thioacetamide liver injury in diabetic rats is due to induced CYP2E1. J. Pharmacol. Exp. Ther..

[cit23] Kim S., Sohn I., Ahn J. I., Lee K. H., Lee Y. S. (2004). Hepatic gene expression profiles in a long-term high-fat diet-induced obesity mouse model. Gene.

[cit24] Osabe M., Sugatani J., Fukuyama T., Ikushiro S., Ikari A., Miwa M. (2008). Expression of hepatic UDP-glucuronosyltransferase 1A1 and 1A6 correlated with increased expression of the nuclear constitutive androstane receptor and peroxisome proliferator-activated receptor alpha in male rats fed a high-fat and high-sucrose diet. Drug Metab. Dispos..

[cit25] Koide C. L., Collier A. C., Berry M. J., Panee J. (2010). The effect of bamboo extract on hepatic biotransforming enzymes-findings from an obese-diabetic mouse model. J. Ethnopharmacol..

[cit26] Sheweita S. A., Newairy A. A., Mansour H. A., Yousef M. I. (2002). Effect of some hypoglycemic herbs on the activity of phase I and II drug-metabolizing enzymes in alloxan-induced diabetic rats. Toxicology.

[cit27] Latha M., Pari L. (2004). Effect of an aqueous extract of *Scoparia dulcis* on blood glucose, plasma insulin and some polyol pathway enzymes in experimental rat diabetes. Braz. J. Med. Biol. Res..

[cit28] Lichtenstein A. H., Schwab U. S. (2000). Relationship of dietary fat to glucose metabolism. Atherosclerosis.

[cit29] Riccardi G., Aggett P., Brighenti F., Delzenne N., Frayn K., Nieuwenhuizen A., Pannemans D., Theis S., Tuijtelaars S., Vessby B. (2004). PASSCLAIM–body weight regulation, insulin sensitivity and diabetes risk. Eur. J. Nutr..

[cit30] Pratchayasakul W., Kerdphoo S., Petsophonsakul P., Pongchaidecha A., Chattipakorn N., Chattipakorn S. C. (2011). Effects of high-fat diet on insulin receptor function in rat hippocampus and the level of neuronal corticosterone. Life Sci..

[cit31] Jeon B. T., Jeong E. A., Shin H. J., Lee Y., Lee D. H., Kim H. J., Kang S. S., Cho G. J., Choi W. S., Roh G. S. (2012). Resveratrol attenuates obesity-associated peripheral and central inflammation and improves memory deficit in mice fed a high-fat diet. Diabetes.

[cit32] McNeilly A. D., Williamson R., Balfour D. J., Stewart C. A., Sutherland C. (2012). A high-fat-diet-induced cognitive deficit in rats that is not prevented by improving insulin sensitivity with metformin. Diabetologia.

[cit33] Pipatpiboon N., Pintana H., Pratchayasakul W., Chattipakorn N., Chattipakorn S. C. (2013). DPP4-inhibitor improves neuronal insulin receptor function, brain mitochondrial function and cognitive function in rats with insulin resistance induced by high-fat diet consumption. Eur. J. Neurosci..

[cit34] Bunn H. F., Higgins P. J. (1981). Reaction of monosaccharides with proteins: possible evolutionary significance. Science.

[cit35] Ahmed N., Thornalley P. J. (2007). Advanced glycation end products: what is their relevance to diabetic complications?. Diabetes, Obes. Metab..

[cit36] Nagarjuna R. V., Nagarathna P. K. M., Divya M. (2013). Evaluation of anti-cancer activity of *Ruellia tuberosa* on EAC induced mammary tumor. International Journal of Pharmacology and Toxicology.

[cit37] Giugliano D., Ceriello A., Paolisso G. (1995). Diabetes mellitus, hypertension, and cardiovascular disease: which role for oxidative stress?. Metabolism.

[cit38] Grankvist K., Marklund S. L., Täljedal I. B. (1981). Cu/Zn-superoxide dismutase, Mn-superoxide dismutase, catalase and glutathione peroxidase in pancreatic islets and other tissues in the mouse. Biochem. J..

[cit39] Andallu B., Varadacharyulu N. C. (2003). Antioxidant of mulberry (*Morus indica* L. cv. Anantha) leaves in streptozocin-diabetic rats. Clin. Chim. Acta.

[cit40] Bhatia S., Shukla R., Madhu S. V., Gambhir J. K., Prabhu K. M. (2003). Antioxidant status, lipid peroxidation and nitric oxide end products in patients of type 2 diabetes mellitus with nephropathy. Clin. Biochem..

[cit41] Seghrouchni I., Drai J., Bannier E., Rivière J., Calmard P., Garcia I., Orgiazzi J., Revol A. (2002). Oxidative stress parameters in type I, type II and insulin-treated type 2 diabetes mellitus, insulin treatment efficiency. Clin. Chim. Acta.

[cit42] Milani E., Nikfar S., Khorasani R., Zamani M. J., Abdollahi M. (2005). Reduction of diabetes-induced oxidative stress by phosphodiesterase inhibitors in rats. Comp. Biochem. Physiol., Part C: Toxicol. Pharmacol..

[cit43] Paolisso G., Gambardella A., Tagliamonte M. R., Saccomanno F., Salvatore T., Gualdiero P., D'Onofrio M. V., Howard B. V. (1996). Does free fatty acid infusion impair insulin action also through an increase in oxidative stress?. J. Clin. Endocrinol. Metab..

[cit44] Robertson G., Leclercq I., Farrell G. C. (2001). Nonalcoholic steatosis and steatohepatitis. II. Cytochrome P-450 enzymes and oxidative stress. Am. J. Physiol.: Gastrointest. Liver Physiol..

